# Differential timing of mitochondrial activation in rat dorsal striatum induced by procedural learning and swimming

**DOI:** 10.3389/fnmol.2024.1495027

**Published:** 2024-12-04

**Authors:** Rogelio Pegueros-Maldonado, Antonio Fuentes-Ibañez, Mónica M. Monroy, Oscar A. Gutiérrez, Norma Serafín, Santiago M. Pech-Pool, Mauricio Díaz-Muñoz, Gina L. Quirarte

**Affiliations:** ^1^Departamento de Neurobiología Conductual y Cognitiva, Instituto de Neurobiología, Universidad Nacional Autónoma de México, Querétaro, Mexico; ^2^Facultad de Química, Universidad Autónoma de Querétaro, Querétaro, Mexico; ^3^Departamento de Neurobiología Celular y Molecular, Instituto de Neurobiología, Universidad Nacional Autónoma de México, Querétaro, Mexico

**Keywords:** cued Morris water maze, consolidation, dorsal striatum, mitochondrial activity, corticosterone, rotenone

## Abstract

Stressful experiences form stronger memories due to enhanced neural plasticity mechanisms linked to glucocorticoid hormones (cortisol in humans, corticosterone in rats). Among other neural structures, the dorsal striatum plays a role in the corticosterone-induced consolidation of stressful memories, particularly in the cued water maze task. Neural plasticity is related to mitochondrial activity due to the relevance of energy production and signaling mechanisms for functional and morphological neuronal adaptations. Corticosterone has been shown to enhance brain mitochondrial activity by activating glucocorticoid receptors. In this context, striatum functions are susceptible to change in relation to mitochondrial responses. Based on this evidence, we hypothesized that training in the cued water maze would induce an increase in corticosterone levels and mitochondrial activity (mitochondrial membrane potential and calcium content) in the dorsal striatum, and that these adaptations might be related to memory consolidation of the task. We used an ELISA assay to evaluate plasma and striatal corticosterone levels; mitochondrial activity was determined with the florescent probes MitoTracker Red (mitochondrial membrane potential) and Rhod-2 (calcium content) in brain slices containing the dorsal striatum of rats trained in the cued water maze and euthanized at different times after training (0.5, 1.5, or 6.0 h). We also analyzed the effect of post-training inhibition of striatal mitochondrial activity by OXPHOS complex 1 inhibitor rotenone, on the consolidation of the cued water maze task. We found that cued water maze training induced an increase in corticosterone levels and a time-dependent elevation of mitochondrial membrane potential and mitochondrial calcium content in the dorsal striatum. Unexpectedly, rotenone administration facilitated the retention test. Altogether, our results suggest that enhanced mitochondrial activity in the dorsal striatum is relevant for cued water maze consolidation. The increase in mitochondrial activity was contextually associated with an elevation of corticosterone in plasma and the dorsal striatum. Additionally, our swimming groups also showed an increase in mitochondrial activity in the dorsal striatum, but with a different pattern, which could suggest a differential functional adaptation in this structure.

## 1 Introduction

Aversive learning experiences are associated with the activation of the hypothalamic-pituitary-adrenal axis, which in turn modulates the release of glucocorticoids, corticosterone (CORT) in rats, and cortisol in humans (de Kloet, 2013). It has been reported that systemic or local injection of CORT enhances memory consolidation in aversive memory paradigms, such as inhibitory avoidance and the Morris water maze (Sandi et al., 1997; Medina et al., 2007). Procedural memories allow subjects to solve tasks using a stimulus-response strategy rather than a spatial strategy in paradigms such as the cued Morris water maze (Rice et al., 2015). Research has shown that the dorsal striatum is required for memory consolidation in this type of task (Miyoshi et al., 2012) and that this consolidation process is partly modulated by CORT, since direct administration enhances memory consolidation (Quirarte et al., 2009).

In several organs, including the brain, glucocorticoids activate two nuclear receptors: mineralocorticoid receptor and glucocorticoid receptor (GR; Reul and de Kloet, 1985). Both receptors act as transcriptional factors that modulate the expression of brain plasticity-related genes (Datson et al., 2008). Apart from its canonical function, GR can also translocate into brain mitochondria (Moutsatsou et al., 2001), modulating mitochondrial membrane potential and thus influencing reactive oxygen species (ROS) production and calcium dynamics. It has been reported that the incubation of neuronal cultures with CORT leads to an increase in these processes, which is dependent on GR activation (Du et al., 2009; Choi et al., 2018). This effect is related to memory formation because synaptic plasticity during memory consolidation is closely related to mitochondrial activity. This includes calcium dynamics and signaling in long-term potentiation (Kim et al., 2011), modulation of the dendritic spine population (Ertürk et al., 2014), and energy production in the soma, axons, and dendrites (Li et al., 2004). The suggestion that CORT could modulate neural plasticity by regulating mitochondrial activity has created new research opportunities. This integration of metabolic changes and brain plastic processes is significant and offers a novel approach to understanding how stressful memories are formed.

To support this idea, it has been reported that striatum-dependent memory is disrupted when mitochondrial activity is compromised (Mu et al., 2011; Chen et al., 2017), and that striatal responses are highly susceptible to mitochondrial alterations (Pickrell et al., 2011). Therefore, it is plausible that, during a stressful memory experience, an increase in CORT levels could induce an adaptation of mitochondrial activity in the dorsal striatum. This adaptation, in turn, could lead to synaptic plasticity events associated with memory consolidation. To assess this hypothesis, we assessed mitochondrial activity using the cationic fluorophores MitoTracker red (mitochondrial membrane potential, ΔΨm) and Rhod-2 (mitochondrial Ca^+2^ content) in dorsal striatum slices of rats trained in the cued water maze and euthanized at different times after training (0.5, 1.5, or 6 h). As a control parameter, we assessed dorsal striatum mitochondrial density by measuring OXPHOS complex IV subunit IV (COX-IV) immunofluorescence in the same experimental protocol, in that regard, we also explored the effect of lessening the mitochondrial capacity of the dorsal striatum during memory consolidation in the cued water maze by injecting rats with rotenone, an inhibitor of the OXPHOS complex I. Lastly, we trained rats in the cued version of the water maze and measured plasma and dorsal striatal levels of CORT.

## 2 Method

### 2.1 Subjects

This project involved 118 male adult Wistar rats (*Rattus norvegicus albinus*, weight: 250–350 g at the time of surgery) obtained from the breeding colony of the Instituto de Neurobiología of the Universidad Nacional Autónoma de México (UNAM) and maintained at the vivarium of our laboratory in individual acrylic home cages (24 × 21 × 45 cm) with *ad libitum* access to food and water as well as a 12/12 h light/dark cycle starting at 07:00 h. Animals were maintained in accordance with Official Mexican Standard NOM-062-ZOO-1999 (NORMA, 2001) and the recommendations of the Guide for the Care and Use of Laboratory Animals of the National Research Council (National Research Council, 2011). The protocols for these experiments were approved by the Ethics Committee of the Instituto de Neurobiología, UNAM. All subjects exposed to the water maze apparatus were handled for 5 min for three days before the experiments.

### 2.2 Cued water maze

The rats were trained in a water maze as previously described (Quirarte et al., 2009). The apparatus consisted in a black circular plastic tank (1.54 m in diameter and 0.60 m in height) filled with water (25 ± 1°C) to a depth of 21 cm, surrounded by black curtains to avoid distal cues. Four starting positions were spaced equally around the pool perimeter, giving four quadrants. The rat was placed in the tank at one of the four designated starting points, facing the wall, and allowed to escape onto a visible platform (12 × 12 cm) marked by a white and green striped cylinder. If subjects missed the platform in the first trial, they were guided to it. The maximum duration of each trial was 60 s. After mounting the platform, the rat remained there for 10 s and then was placed in a holding box for 30 s until the next trial. A total of eight trials were carried out. The platform was moved to a different location on each trial, such that each of the four quadrants contained the escape platform twice. The locations of the starting points were organized according to the distance to the escape platform (i.e., proximal or distal) and the location of the platform relative to the starting point (i.e., left or right) to be counterbalanced across trials. Forty-eight h after the training session, each rat was given one retention trial. Escape latencies were measured with Any-maze software (Stoelting Co., Illinois, USA). For the mitochondrial probes and immunohistochemistry measurements, a swimming group was added as a motor activity control to assess stress and motor components. The subjects in this group swam freely for eight trials in the water maze tank without the platform for the mean time the trained groups performed each trial of the task (from one to eight trials: 48, 36, 20, 23, 25, 15, 21, and 20 s). Latencies were used as an indication of memory; shorter latencies were interpreted as a more efficient memory.

### 2.3 Tissue processing

After the behavioral procedures, all animals were quickly euthanized without anesthesia by decapitation with a guillotine, always assuring to avoid probable aversive stimuli during the process. For the mitochondrial activity probes and immunohistochemistry, independent groups of trained or swimming rats were sacrificed at different time points: 0.5, 1.5, or 6.0 h after training. Another control group (caged group) was added to all experimental procedures. The rats in this group were kept in their cage until euthanasia.

For mitochondrial activity measurements, brains were processed immediately after euthanasia, and slices corresponding to the dorsal striatum were collected for staining. For immunohistochemistry and corticosterone measurement, brains were frozen in isopentane (C5H12) cooled with dry ice (CO_2_) for later use. In all experiments, we used an extraction range of 0.0 to 2.0 mm from Bregma (Paxinos and Watson, 2007).

### 2.4 Immunohistochemistry

To evaluate mitochondrial density, we measured the presence of COX-IV (Choi et al., 2018). Brain sections (16 μm thick) were obtained from frozen brains using a cryostat and immediately fixed in 4% paraformaldehyde for 20 min.

Epitope exposure was enhanced by incubation in sodium citrate at 70–80°C for 20 min. The following antibodies were used: mouse anti-COX-IV primary antibody at 1:500 dilution (Abcam, ab33985) and the Biotin-Streptavidin system (Berghorn et al., 1994) including the biotinylated anti-mouse secondary antibody (Thermo Fisher S11227; 1:200 dilution, for 2 h) and streptavidin coupled to the Alexa-594 fluorophore (Thermo Fisher 31806; 1:400 dilution, for 2 h). Washing steps were done with the buffers TBS1X and TTBS1X (0.05% Triton). Non-specific binding sites were blocked using 5% non-fat dry milk (Bio-Rad, Hercules, CA, USA). Tissues were treated with Sudan Black (0.01% in 70% ethanol) for the reduction of autofluorescence, and cell nuclei were stained with DAPI (Sigma-Aldrich, D9542).

### 2.5 Mitochondrial activity probes

For the measurement of mitochondrial activity, we used cationic fluorophores to label mitochondrial membrane potential and mitochondrial Ca^+2^ content in 1 mm-thick dorsal striatum slices that were obtained using a steel brain matrix immediately after euthanasia. The slices were counterbalanced and placed in Ringer solution (155 mM NaCl, 4.5 mM KCl, 1.9 mM NaHCO_3_, 2.4 mM CaCl_2_, 10 mM glucose, 2 mM MgCl_2_, 5 mM HEPES, pH 7.5; modified from Bindokas et al., 1998). One hemisphere was incubated with 0.5 μM MitoTracker Red CMXRos (Thermo Fisher, M7512) to measure mitochondrial membrane potential, and the other hemisphere was incubated with 2–3 μM DyhidroRhod-2 prepared with NaBH4 according to the manufacturer's instructions (Thermo Fisher, R1245MP) to measure mitochondrial Ca^+2^ content.

For fluorophore staining, brain slices were first equilibrated with Ringer solution at room temperature for 30 min and then moved to another container with the fluorophores in Ringer solution. The container was kept at a constant temperature of 37°C, in the dark, and with constant oxygenation for 1 h for fluorophore loading (adapted from Johnson et al., 2018). When finished, the tissues were fixed in 4% paraformaldehyde for 24 h. The tissues were subsequently cryoprotected using 10%, 20%, and 30% sucrose (Vázquez-Martínez et al., 2019), and then cut to obtain 16 μm sections. After this step, tissues were treated with Sudan Black (0.01% in 70% ethanol) and Sytox Green (600 nM; Thermo Fisher, S7020) for the reduction of autofluorescence (He et al., 2023) and nuclear labeling, respectively. Washes were done with TBS1X.

### 2.6 Confocal imaging and analysis

**Figure 1:**
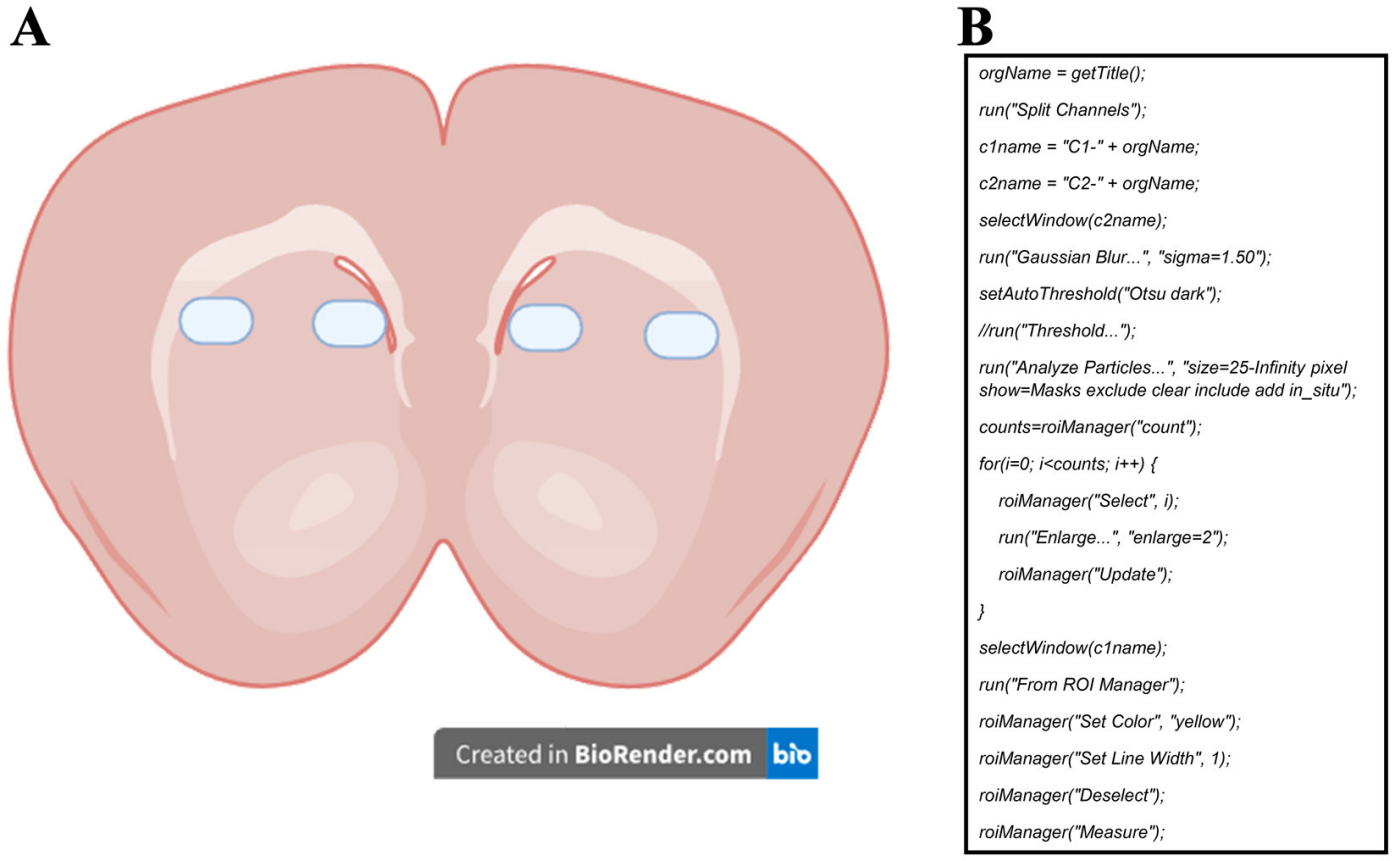
**(A)** Representative diagram of the sampling zones for the micrographs used to measure mitochondrial activity probes and COX-IV immunoreactivity. **(B)** ImageJ macro developed to measure the mean fluorescence of each cell in all micrographs. Channel 1 (c1) was set for the dye of interest (MitoTracker Red, Rhod-2, or COX-IV) to measure mean fluorescence, while channel 2 (c2) was set for SYTOX Green or DAPI staining and used for mask area selection.

Micrographs were captured using an LSM 780 confocal microscope (Carl Zeiss, Germany) at 40X magnification and a single focal plane. For mitochondrial activity probes, a total of 18 to 30 micrographs were acquired (6 per animal, from one hemisphere, counterbalancing MitoTracker Red and Rhod-2). For immunohistochemistry samples, 48 to 60 micrographs were obtained (12 per animal, covering both hemispheres). Figure 1A illustrates the selected areas for image acquisition. Along the anterior-posterior brain axis, 2 micrographs were taken from each of 3 slides, spaced 30 μm apart.

The micrographs were post-processed and analyzed with ImageJ software (National Institutes of Health and the Laboratory for Optical and Computational Instrumentation, USA). We quantified the proportion of positive cells over the total number of cells for each marker. The method consisted of a semi-automatic analysis using nucleus signals as area masks (Miura et al., 2020). These area masks were later enlarged to measure the mean gray value of the marker of interest in each cell (Figure 1B). Measurements were registered in a database and used to select a “positive” cell reference value based on a clear visualization of the staining color near the nucleus. This value was later used in all images to count the number of cells with values greater than the reference, thus, considered positive cells. We must emphasize that the positive reference value was always picked from images of the caged group to ensure measuring from a “basal” level of the dyes. The values of positive cells were later divided by the total number of cells to calculate the positive cell ratio.

### 2.7 Stereotaxic surgery and cannula implantation

Rats were anesthetized with sodium pentobarbital (Pisabarbital, 50 mg/kg, ip) and then secured in a stereotaxic frame (Stoelting, Co., llinois, USA). Bilateral guide cannulae (11 mm, 23 gauge) were carefully inserted into the dorsal striatum following the coordinates outlined by Paxinos and Watson (2007): anteroposterior: +0.4 mm from Bregma, mediolateral: ±3.2 mm from midline, dorsoventral: 4.2 mm below the skull surface. Stylets (11 mm long) were utilized to maintain cannula patency and removed only for drug administration and handling sessions. Rats were given at least five days to recover before the behavioral procedures.

### 2.8 Drug and infusion procedures

We bilaterally administered rotenone (0.8 or 1.2 μg/μL, 2.02 mM and 3.03 mM, respectively; ALX-350-360, Enzo Life Sciences, Farmingdale, NY, USA) or vehicle into the dorsal striatum immediately after the training session in the cued water maze. Rotenone was diluted in a 1:1:2.6 dilution of DMSO, polyethylene glycol, and saline solution, modified from Mulcahy et al. (2011).

Injection needles were inserted 1.0 mm beyond the cannula tips, and a 0.5 μL injection volume per hemisphere was infused at a rate of 0.5 μL/min using an automated micro infusion pump (WPI, model 220i). The injection needles were left inside the cannula for an additional 60 s to ensure maximum drug diffusion.

### 2.9 Corticosterone measurement

CORT levels in plasma and dorsal striatum homogenates were measured using an ELISA kit following the manufacturer's instructions (Abcam, #ab108821). Fifteen min after training, subjects were euthanized, and we obtained the trunk's blood, from which plasma was obtained by centrifugation at 3,000 g for 10 min. For the dorsal striatum, we extracted tissue from frozen brains using a steel brain matrix. The tissue was then dissected, weighted, and mixed with the kit's diluent buffer, and centrifuged at 5,000 g for 15 min as previously described in Pegueros-Maldonado et al. (2024). The obtained data from the dorsal striatum homogenate was referred to tissue weight to express results in ng/mg.

### 2.10 Statistical analysis

Training escape latencies were analyzed with a repeated measures two-way ANOVA, while the retention test and CORT levels were compared with a one-way ANOVA. Both tests were further analyzed with a Tukey *post-hoc* test, apart from the trained group used for ELISA measurements, which was analyzed using a repeated measures ANOVA and a Tukey *post*-*hoc* test. Positive cell ratios of the mitochondrial probes (MitoTracker red and Rhod-2), as well as COX-IV immunohistochemistry, were compared with the caged group with an unpaired *t*-test. All data were analyzed for normality with a Shapiro-Wilks test. Analyses were performed with GraphPad Prism 8. *p* < 0.05 was considered statistically significant. For some behavioral results obtained from the ANOVAs, we compared the first and last trial of the training session with a *t*-test to corroborate that a strong tendency could show statistical differences masked by the grouped analysis.

## 3 Results

### 3.1 Striatal mitochondrial activity increases during memory consolidation in a time-dependent manner

Brain plasticity processes during memory consolidation require a high metabolic demand, which is mainly met by mitochondrial function. To explore if the cued water maze training could increase mitochondrial activity in the dorsal striatum, we analyzed the fluorescent signals associated with mitochondrial density, mitochondrial membrane potential, and mitochondrial calcium content at different times after training (0.5, 1.5, or 6.0 h).

**Figure 2:**
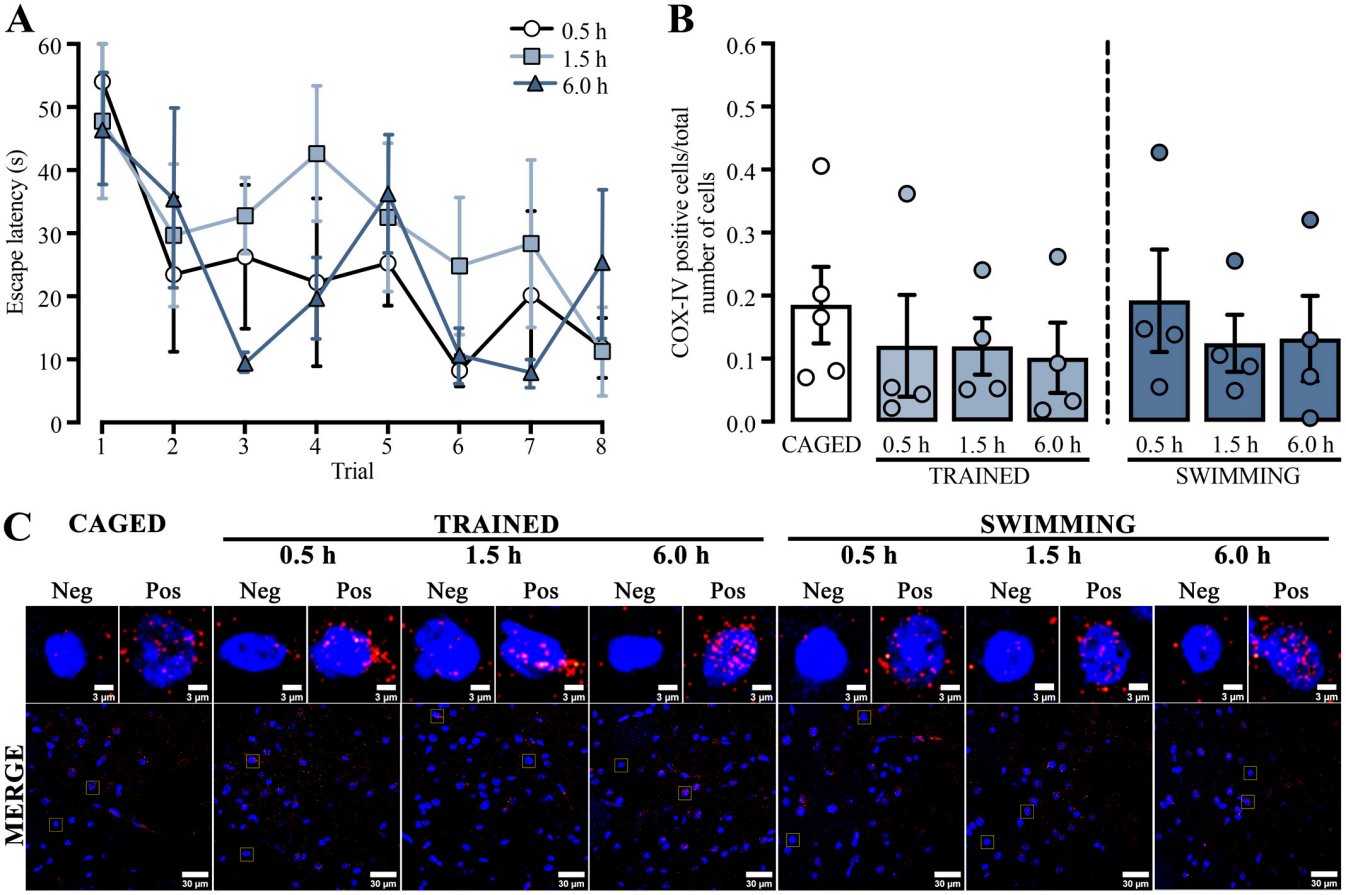
**(A)** Cued water maze training data of rats that were subsequently euthanized at 0.5, 1.5, or 6.0 h post-training, analyzed for mitochondrial density using immunohistochemistry. *n* = 4. **(B)** Ratio of COX-IV-positive cells; a *t*-test showed no statistical differences. *n* = 4–5. Data are presented as mean ± SEM, with small circles representing individual values. **(C)** Representative micrographs of the dorsal striatum from experimental and control groups, showing COX-IV fluorescence in red and the nuclei in blue.

We used a repeated measures two-way ANOVA to compare the training escape latencies of the subjects in which mitochondrial density in the dorsal striatum was analyzed by immunohistochemistry to assess possible changes in mitochondrion presence (Figure 2A). Differences were detected in the trial factor [*F*_(3.60, 32.43)_ = 4.708, *p* = 0.005] but not in the group factor [*F*_(2, 9)_ = 0.777, *p* = 0.48] or the interaction [*F*_(14, 63)_ = 0.881, *p* = 0.58]. A Tukey *post-hoc* test did not yield statistical differences between the first and last trials in any group. A subsequent statistical analysis of this comparison showed a decrease in the last trial for the group euthanized 0.5 h after training [t_(6)_ = 5.516, *p* = 0.001] and the group evaluated at 1.5 h [t_(6)_ = 2.585, *p* = 0.04], but not for the 6.0 h group [t_(6)_ = 1.418, *p* = 0.20].

These results indicated that the subjects sacrificed at 0.5 h and 1.5 h learned the task adequately. Moreover, the group sacrificed 6.0 h after training did not acquire the task similarly to the other groups. It is plausible that the results were affected by the small number of subjects in this group, as four subjects could be insufficient for robust behavioral analysis.

To evaluate if the changes in mitochondrial activity could only be related to functional processes, we analyzed mitochondrial density (Figure 2C) by comparing the proportion of positive cells for COX-IV immunoreactivity and the total number of cells in the dorsal striatum (Figure 2B). The data showed no significant differences in any group or condition (*p* > 0.05). These results suggest that neither training nor swimming experimental conditions induced changes in mitochondrial density in the dorsal striatum.

**Figure 3:**
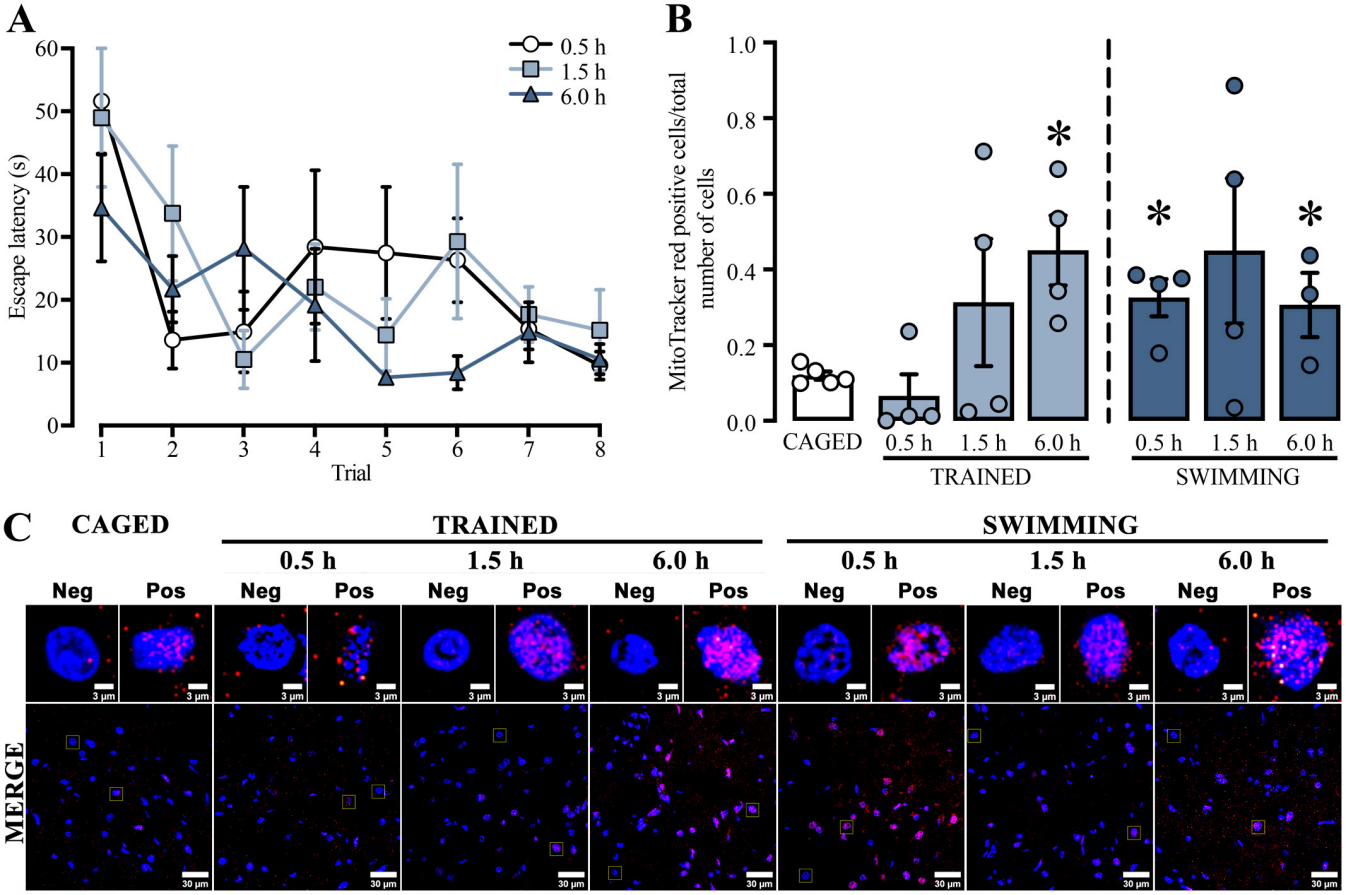
**(A)** Cued water maze training data of subjects that were subsequently euthanized at 0.5, 1.5, or 6.0 h post-training analyzed with the mitochondrial activity probes. *n* = 3–4. **(B)** Ratio of MitoTracker red-positive cells; a *t*-test indicated statistical differences compare to the CAGED group, (*) *p* < 0.05. *n* = 3–5. Data are presented mean ± SEM, with small circles representing individual values. **(C)** Representative micrographs of the dorsal striatum from experimental and control groups, showing MitoTracker fluorescence in red and nuclei in blue.

For the next experiment, we used a repeated measures two-way ANOVA to analyze the training latencies of the subjects that were subsequently assessed with the mitochondrial activity probes (Figure 3A). The analysis showed significant differences in the trial factor [*F*_(3.41, 30.72)_ = 5.342, *p* = 0.003], but not in the group factor [*F*_(2, 9)_ = 2.660, *p* = 0.12] or the interaction [*F*_(14, 63)_ = 1.110, *p* = 0.36]. The Tukey *post-hoc* test did not yield statistical differences between the first and last trials in any group. A subsequent statistical analysis of this exact comparison indicated a decrease in the latencies in the last trial of all the euthanasia temporalities: 0.5 h group [*t*_(6)_ = 4.854, *p* = 0.002], 1.5 h group [*t*_(6)_ = 2.646, *p* = 0.03], and the 6.0 h group [*t*_(6)_ = 2.724, *p* = 0.03]. These results indicated that the subjects sacrificed at 0.5 h, 1.5 h, or 6.0 h post-training performed similarly in the cued water maze training session.

To study whether training in the cued water maze influenced mitochondrial membrane potential (Figure 3C), ΔΨm was determined by measuring the ratio of MitoTracker red-positive cells over the total number of cells in the dorsal striatum (Figure 3B).

All statistical comparisons were made with the basal levels of the caged group. We found a significant time-dependent increase in the trained and swimming groups. The trained groups did not show an increase at 0.5 h [*t*_(7)_ = 1.047, *p* = 0.330] or 1.5 h [*t*_(7)_ = 1.30, *p* = 0.23] after training. However, a marked increase was observed at 6.0 h [*t*_(7)_ = 4.037, *p* = 0.005]. The swimming groups showed a slightly different dynamic. First, there was a significant increment at 0.5 h [*t*_(7)_ = 4.584, *p* = 0.002], followed by a rising tendency at 1.5 h [*t*_(7)_ = 1.949, *p* = 0.09], and then a significant increase at 6.0 h [*t*_(6)_ = 2.938, *p* = 0.02]. These findings suggest that both training in the cued water maze and the action of swimming induce a time-dependent increase in mitochondrial membrane potential in the dorsal striatum, although with different temporal dynamics.

**Figure 4:**
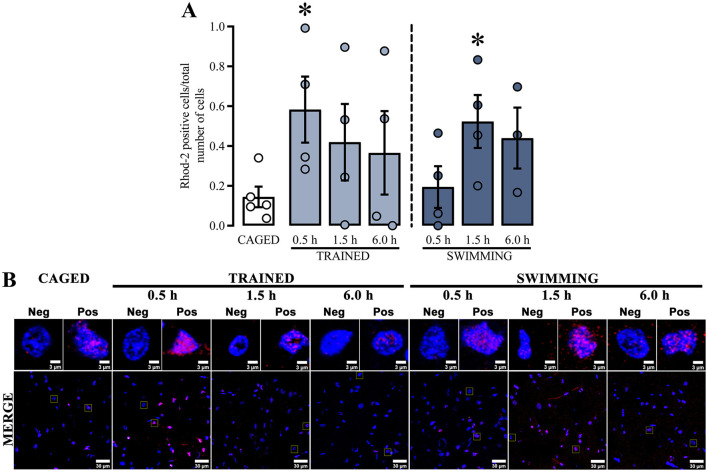
**(A)** Ratio Rhod-2-positive cells; a *t*-test indicated statistical differences compare to the CAGED group; (*) *p* < 0.05. *n* = 3–5. Data represent mean ± SEM, with small circles representing individual values. **(B)** Representative micrographs of the dorsal striatum of experimental and control groups, Rhod-2 fluorescence in red and the nuclei in blue.

Regarding calcium dynamics, Ca^+2^ content (Figure 4A) was determined by measuring the ratio of Rhod-2 positive cells and the total number of cells in the dorsal striatum. The positive cell ratios from all groups were compared with the basal levels of the caged group (Figure 4B). Similar to the mitochondrial membrane potential results, the Rhod-2 positive cell ratio increased at specific times, which were also significantly different in trained and swimming subjects. In the case of trained subjects, a statistical increase was observed at 0.5 h [*t*_(7)_ = 2.793, *p* = 0.02], but not at 1.5 h [*t*_(7)_ = 1.537, *p* = 0.16] or 6.0 h [*t*_(7)_ = 1.146, *p* = 0.28] post-training. On the other hand, the swimming subjects did not show significant differences at 0.5 h [*t*_(7)_ = 0.4567, *p* = 0.66] or 6.0 h [*t*_(6)_ = 2.249, *p* = 0.06], but there was a significant increase in at 1.5 h [*t*_(7)_ = 2.904, *p* = 0.02] after training. These data suggest that both training and swimming induce a time-dependent increase in mitochondrial Ca^+2^ content after training in the cued water maze task, although with different temporal dynamics.

### 3.2 Rotenone treatment in the dorsal striatum induces memory facilitation

**Figure 5:**
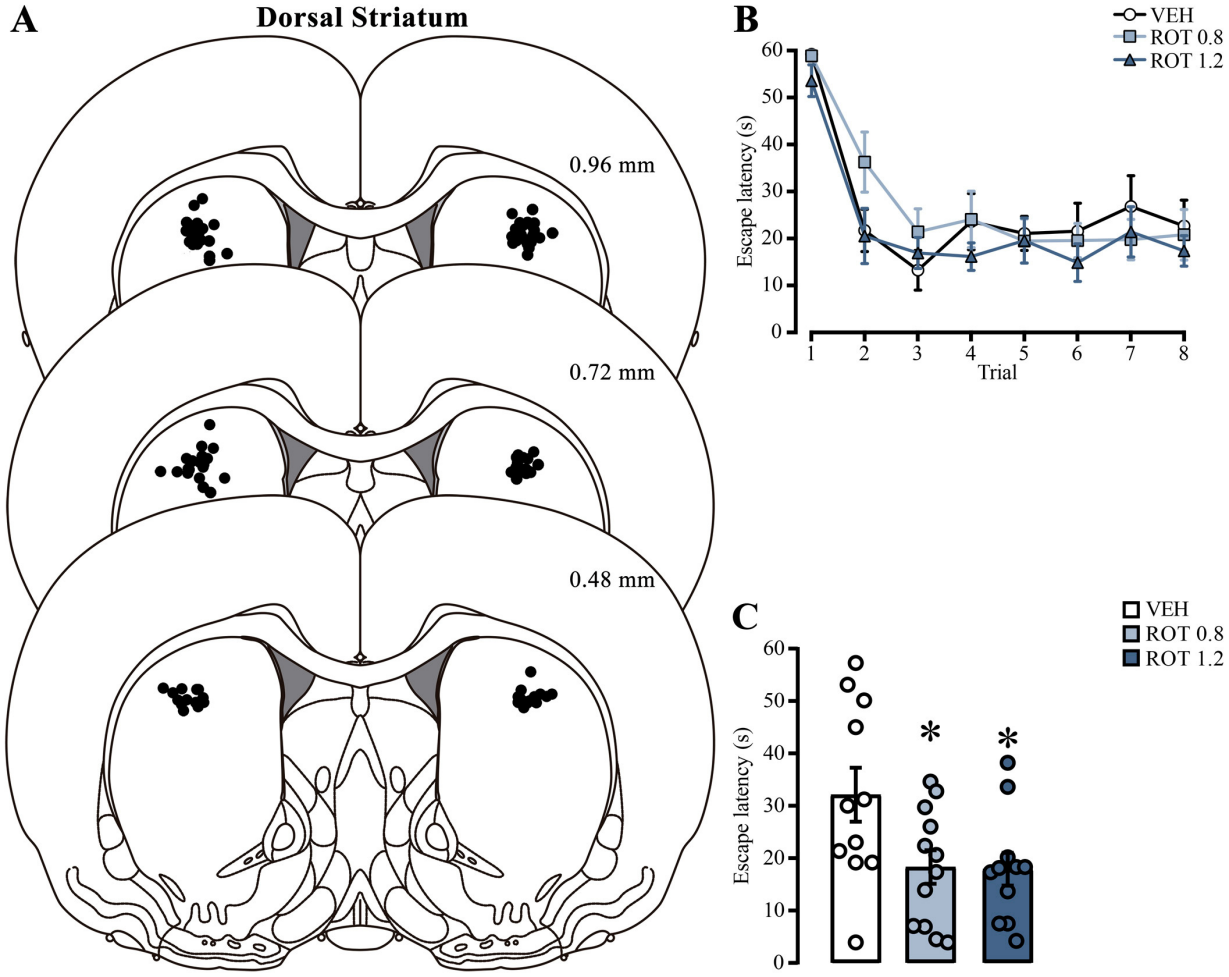
Effect of rotenone injection into the dorsal striatum on memory consolidation of the cued water maze. **(A)** Representative diagrams showing injection sites in the dorsal striatum for all groups; each black dot represents the cannula tip of each rat. **(B)** Escape latencies during training trials; statistical analyses showed no significant differences among the groups. **(C)** Retention latencies; statistical analyses indicated significant differences compared to the vehicle group (*) *p* < 0.05. *n* = 11–12. Data are presented as mean ± SEM, with mall circles representing individual values. VEH, vehicle; ROT, rotenone.

To assess if mitochondrial activity is a relevant factor for memory consolidation, we analyzed the effect of inhibiting the mitochondrial OXPHOS complex I by direct injection of rotenone into the dorsal striatum immediately after training. We only analyzed data from animals with correct cannula placement in the dorsal striatum (Figure 5A). The escape latencies of each subject were evaluated on the day of training (Figure 5B). Two-way ANOVA analysis did not show differences in the treatment factor [*F*_(2, 248)_ = 2.423, *p* = 0.09] or interaction [*F*_(14, 248)_ = 0.6807, *p* = 0.79], indicating that all groups were in similar conditions before rotenone treatment. Statistical differences were detected in the trial factor [*F*_(7, 248)_ = 23.44, *p* < 0.001], with *post-hoc* comparisons showing lower latencies in the last trial than in the first one, which indicates learning in the task (*p* < 0.001, in all group comparisons). Forty-eight h later, we measured retention memory (Figure 5C) and found an unexpected memory facilitation effect [one-way ANOVA; *F*_(2, 31)_ = 4.239, *p* = 0.02] because rats with 0.8 or 1.2 μg rotenone showed lower escape latencies than those treated with vehicle (*p* = 0.04, for both comparisons). These results suggest that intra-striatal rotenone treatment enhances memory consolidation of the cued water maze task. To confirm that rotenone can affect mitochondrial activity, we tested its efficacy by directly incubating brain slices (500–600 nM) containing the dorsal striatum in a counterbalanced manner and measuring its effect on ΔΨm. We observed a significant reduction [*t*_(6)_ = 2.775, *p* = 0.03] in the proportion of MitoTracker red-positive cells in rotenone-treated hemispheres (Supplementary Figure 1).

### 3.3 Cued water maze training and swimming induced an increase in plasma and dorsal striatum corticosterone levels

Swimming in the cued water maze represents a stressful event, and research has demonstrated that CORT is an important modulator for mitochondrial activity. We explored whether CORT levels in our experimental conditions (15 min after training or swimming) were associated with the mitochondrial changes we detected.

**Figure 6:**
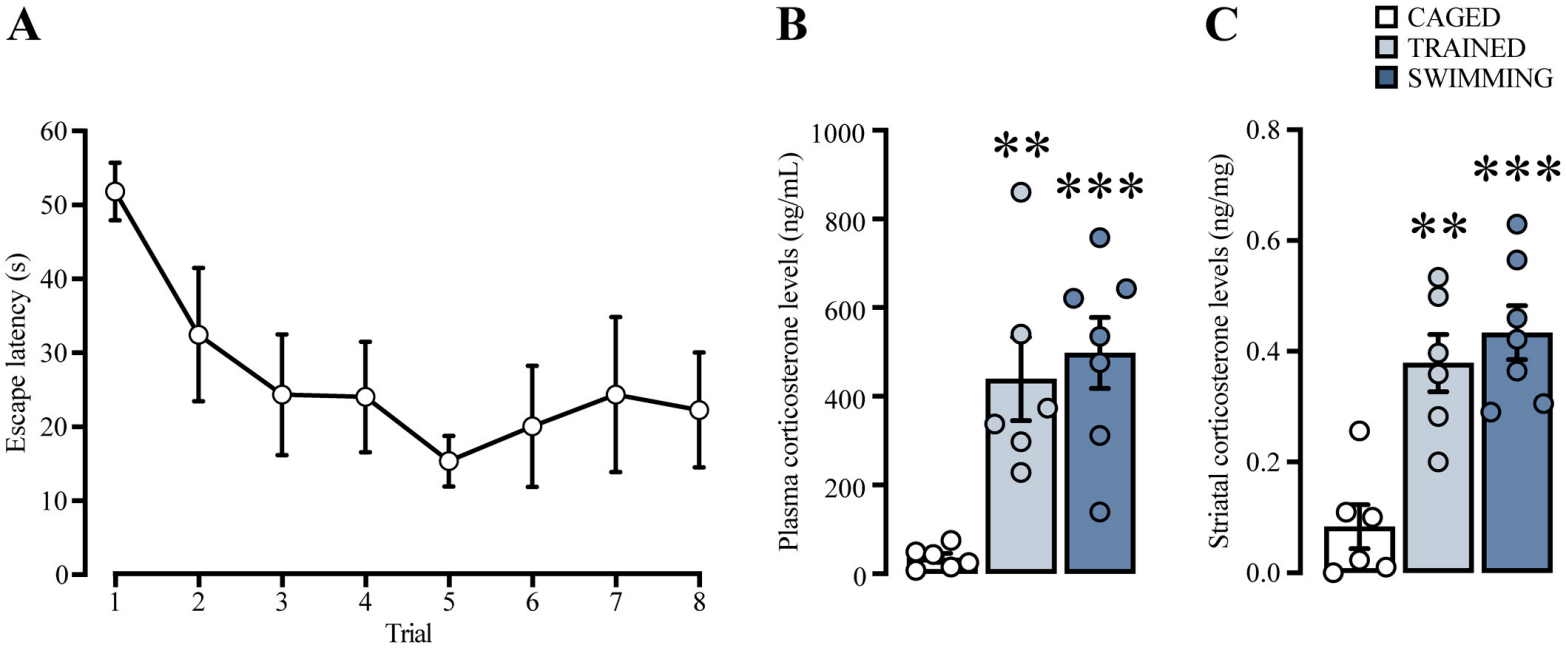
**(A)** Learning curve of a group of rats trained in cue water maze, analyzed for plasma and striatal corticosterone levels. *n* = 6. Cued water maze training and swimming increased corticosterone levels in plasma **(B)** and in striatum **(C)**. The statistical analyses showed significant differences between the trained and swimming groups compared to the caged group, (**) *p* < 0.01. (***) *p* < 0.001. *n* = 6–7. Data are presented as mean ± SEM.

We analyzed the escape latencies of the subjects trained in the cued water maze (Figure 6A). Repeated measures ANOVA did not indicate statistical differences between trials [*F*_(2.669, 13.34)_ = 1.785, *p* = 0.20]. However, a posterior analysis with a paired *t*-test indicated statistically significant lower latencies in the last trial in comparison with the first [*t*_(5)_ = 2.62, *p* = 0.04], suggesting that these subjects as a group were able to learn the task.

Plasma and dorsal striatal homogenate CORT levels (Figures 6B, C) were compared using a one-way ANOVA and Tukey's *post-hoc* test. In the case of plasma CORT levels, there was a significant increase [*F*_(2, 16)_ = 11.72, *p* < 0.001] in the trained (*p* = 0.004) and swimming groups (*p* < 0.001) in comparison with the caged group. On the other hand, CORT levels in the dorsal striatum homogenate also showed statistical differences [*F*_(2, 16)_ = 15.73, *p* < 0.001], with a marked increase in the trained (*p* = 0.001) and swimming groups (*p* < 0.001) in comparison with the caged group. These results suggest that, in our experimental conditions, CORT levels increased in both plasma and dorsal striatum 15 min after training and swimming. These results confirmed that CORT is a robust presence variable in our experimental conditions that could be associated with the consolidation process.

## 4 Discussion

This study aimed to explore if the cued water maze training paradigm or its associated stress could induce adaptations in the mitochondrial activity of the dorsal striatum. We explored the dynamic of mitochondrial membrane potential and Ca^+2^ content. In order to discard a possible variability caused by fission-fusion mitochondrial processes, we first assessed the mitochondrial density obtained from the analysis of COX-IV immunoreactivity. We did not find differences in any group, suggesting that the changes detected in mitochondrial Δψ and Ca^+2^ could not be directly related to a change in the mitochondrial presence but rather to a possible functional adaptation. However, our results do not agree with literature reporting that a depolarizing stimulus in neurons of hippocampal cultures could induce an increase in mitochondrial density (Li et al., 2004). A possible explanation for these contrasting results could be related to our experimental approach, since for our sampling for positive cells we used the nuclei as the initial area of selection, and even though we enlarged the area to measure the immunoreactivity, we may not have found changes in mitochondrial density because this phenomenon has been shown to be more prominent in dendritic spines (Li et al., 2004).

Regarding the mitochondrial activity results, we found a significant increase for both trained and swimming subjects. These findings are relevant, because it has been reported that ΔΨm increases after synaptic excitation (Bindokas et al., 1998) and is important for plasticity mechanisms, such as ATP production. ATP production is a pivotal factor in various cellular processes like axonal vesicle transport (Verstreken et al., 2005), as well as in synaptic pruning, through the caspase signaling pathway (Ertürk et al., 2014). As for all these possible mechanisms, the fact that we encountered a differential time increase in the trained and swimming groups could indicate that mitochondrial membrane potential engages differential processes in our experimental conditions.

In terms of Ca^2+^ content, we also observed an increment of this cation in the trained and swimming groups. Mitochondrial Ca^2+^ dynamics are important for maintaining cellular homeostasis because mitochondria are one of the main regulators of intracellular Ca^2+^ levels. In addition, intramitochondrial Ca^2+^ activates enzymes of the Krebs cycle, favoring ATP production (Denton, 2009). In a neuronal context, it has been reported that mitochondrial Ca^2+^ dynamics is pivotal in synaptic plasticity mechanisms since the blockade of the mitochondrial Ca^2+^ uniporter with Ru360 inhibits the induction of long-term potentiation in spinal cord neurons (Kim et al., 2011). In other reports, it has been shown that mitochondrial activity can be modulated by glutamate transmission, which is also relevant for the correct functioning of the dorsal striatum (Paraskevopoulou et al., 2019). Interestingly, the striatum is strongly innervated by glutamate afferents from almost all cortex areas, and this neurotransmitter may increase mitochondrial respiration (Jekabsons and Nicholls, 2004). This phenomenon may occur because glutamate increases intracellular Ca^2+^ (Beltran-Parrazal et al., 2006), promoting an increase in mitochondrial Ca^2+^ and potentiating mitochondrial activity. Therefore, it is possible that the motor and somatosensory inputs from cortex structures could increase glutamate transmission in the dorsal striatum, hereby enhancing mitochondrial activity through this mechanism.

Our divergent findings with mitochondrial probes for the trained and swimming groups align with those of Chen et al. (2017), who reported that a striatal-specific knockout of an OXPHOS complex I subunit impaired motor performance but did not affect stimulus-response learning, suggesting different mitochondrial adaptations for each process. Similarly, Cid-Duarte et al. (2020) found that hippocampal neural metabolic activity was enhanced in a spatially trained group, whereas the swimming group primarily showed metabolic activity associated with motor performance. The dorsal striatum supports both motor function and stimulus-response learning, with the latter involving a two-phase process: an initial motivational phase and a subsequent phase of behavioral habituation, where repeated behavior becomes habitual (Cataldi et al., 2022). Our findings for trained and swimming groups may thus reflect different functional engagements within the dorsal striatum. Further studies are warranted to examine this intriguing functional dichotomy and its temporal dynamics.

Regarding memory in the cued water maze paradigm, we found an unexpected memory facilitation effect associated with the rotenone treatment. Miyoshi et al. (2012) reported that the dorsal striatum is needed for the cued water maze consolidation process. On the other hand, it has been shown that rotenone could be a negative factor for memory and motor skill mechanisms (Mulcahy et al., 2011; Moreira et al., 2012). This divergence of results may be due to the intrinsic signaling pathways of the ROS produced during the elevation of mitochondrial activity. In that sense, it has been reported that incubation with rotenone elevates ROS production in neuronal dopaminergic cell cultures (Radad et al., 2006). This evidence is relevant since ROS also functions as signaling activators, enhancing enzyme activity of PKA, PKC, and ERK (Kim et al., 2011). It is important to emphasize that the PKA-ERK pathway could be a pivotal factor for memory facilitation in the hippocampus because its downstream activity leads to synaptic functional changes (Guerra et al., 2011), being this pathway also a relevant signaling mechanism for striatal plasticity (Cerovic et al., 2013). This hypothesis is also linked to the interpretation of our mitochondrial probes results, since it is plausible that a glutamate-induced striatal mitochondrial Ca^+2^ content and rotenone blockade of complex I act in synergy to increment ROS production to activate the PKA-ERK pathway and thus induce memory facilitation. Deepening into rotenone effect on mitochondrial activity and exploring ROS levels with fluorophores and then ROS signaling after training could be a valuable approach to deepen our knowledge about these putative mechanisms.

Another relevant aspect for the cued water maze consolidation is its correlation with CORT levels. In this work, we found that both training and swimming groups increased CORT in the plasma and dorsal striatum. These results agree with evidence that swimming acts as a stressor by itself (Harrison et al., 2009) and increases CORT levels in both the hippocampus and striatum (Droste et al., 2008). CORT actions are closely related to mitochondrial mechanisms, and it has been shown that the incubation with CORT potentiates both ΔΨm and Ca^+2^ content (Du et al., 2009; Choi et al., 2018) in neuronal tissue and cultures. This effect is explained by GR translocation into the mitochondria (Du et al., 2009) and by GR association with other proteins, such as VDAC, in the mitochondrial outer membrane (Choi et al., 2018). Another interesting piece of literature is that CORT can directly affect mitochondrial activity; for example, in hypothalamic cell cultures, this hormone decreases ATP levels independently of GR activation. This effect was attributed to the inhibition of complex I (Fujita et al., 2009), since rotenone also inhibits complex I, it is plausible that this mechanism is related to our mitochondrial probes and behavioral results.

In summary, our findings demonstrated that training and swimming in the cued water maze increase mitochondrial activity, which in turn seems to modulate this structure, as seen by the facilitation outcome of the putative mitochondrial OXPHOS complex I inhibition by rotenone. This suggests that mitochondrial activity in the dorsal striatum is closely related to consolidation. Given these observations, we conclude that mitochondrial activity is an important factor for memory consolidation and motor performance in the dorsal striatum, and that these processes are at least contextually associated with an elevation of CORT.

More studies are needed to better understand the dynamics and functional importance of mitochondrial activity in the dorsal striatum. This research should use different time points and experimental conditions, assess the relevance of glutamate and other factors, and explore the signaling pathways associated with ROS levels that may be related to CORT mechanisms and other modulators.

## Data Availability

The raw data supporting the conclusions of this article will be made available by the authors, without undue reservation.

## References

[B1] Beltran-Parrazal L. López-Valdés H. E. Brennan K. C. Díaz-Muñoz M. Vellis J. d. Charles A. C. (2006). Mitochondrial transport in processes of cortical neurons is independent of intracellular calcium. Am. J. Physiol. Cell Physiol. 291, C1193–C1197. 10.1152/ajpcell.00230.2006 16885395

[B2] Berghorn K. A. Bonnett J. H. Hoffman G. E. (1994). cFos immunoreactivity is enhanced with biotin amplification. J. Histochem. Cytochem. 42, 1635–1642. 10.1177/42.12.7983364 7983364

[B3] Bindokas V. P. Lee C. C. Colmers W. F. Miller R. J. (1998). Changes in mitochondrial function resulting from synaptic activity in the rat hippocampal slice. J. Neurosci. 18, 4570–4587. 10.1523/JNEUROSCI.18-12-04570.1998 9614233 PMC6792701

[B4] Cataldi S. Stanley A. T. Miniaci M. C. Sulzer D. (2022). Interpreting the role of the striatum during multiple phases of motor learning. FEBS J. 289, 2263–2281. 10.1111/febs.15908 33977645 PMC8590810

[B5] Cerovic M. d'Isa R. Tonini R. Brambilla R. (2013). Molecular and cellular mechanisms of dopamine-mediated behavioral plasticity in the striatum. Neurobiol. Learn. Mem. 105, 63–80. 10.1016/j.nlm.2013.06.013 23827407

[B6] Chen B. Hui J. Montgomery K. S. Gella A. Bolea I. Sanz E.. (2017). Loss of mitochondrial Ndufs4 in striatal medium spiny neurons mediates progressive motor impairment in a mouse model of leigh syndrome. Front. Mol. Neurosci. 10. 10.3389/fnmol.2017.00265 28883788 PMC5573716

[B7] Choi G. E. Oh J. Y. Lee H. J. Chae C. W. Kim J. S. Jung Y. H.. (2018). Glucocorticoid-mediated ER-mitochondria contacts reduce AMPA receptor and mitochondria trafficking into cell terminus via microtubule destabilization. Cell Death Dis. 9:1137. 10.1038/s41419-018-1172-y 30429451 PMC6235892

[B8] Cid-Duarte S. Gutiérrez-Menéndez A. Zorzo C. Arias J. L. Méndez M. (2020). The swimming control group in spatial reference memory task: analysis of its motor cortex activity. Arch. Ital. Biol. 158, 45–56. 10.12871/00039829202022 33462798

[B9] Datson N. A. Morsink M. C. Meijer O. C. de Kloet E. R. (2008). Central corticosteroid actions: search for gene targets. Eur. J. Pharmacol. 583, 272–289. 10.1016/j.ejphar.2007.11.070 18295201

[B10] de Kloet E. R. (2013). Functional profile of the binary brain corticosteroid receptor system: mediating, multitasking, coordinating, integrating. Eur. J. Pharmacol. 719, 53–62. 10.1016/j.ejphar.2013.04.053 23876452

[B11] Denton R. M. (2009). Regulation of mitochondrial dehydrogenases by calcium ions. Biochim. Biophys. Acta Bioenerg. 1787, 1309–1316. 10.1016/j.bbabio.2009.01.005 19413950

[B12] Droste S. K. de Groote L. Atkinson H. C. Lightman S. L. Reul J. M. Linthorst A. C. (2008). Corticosterone levels in the brain show a distinct ultradian rhythm but a delayed response to forced swim stress. Endocrinology 149, 3244–3253. 10.1210/en.2008-0103 18356272

[B13] Du J. Wang Y. Hunter R. Wei Y. Blumenthal R. Falke C.. (2009). Dynamic regulation of mitochondrial function by glucocorticoids. Proc. Natl. Acad. Sci. U. S. A. 106, 3543–3548. 10.1073/pnas.0812671106 19202080 PMC2637276

[B14] Ertürk A. Wang Y. Sheng M. (2014). Local pruning of dendrites and spines by caspase-3-dependent and proteasome-limited mechanisms. J. Neurosci. 34, 1672–1688. 10.1523/JNEUROSCI.3121-13.2014 24478350 PMC6827581

[B15] Fujita C. Ichikawa F. Teratani T. Murakami G. Okada T. Shinohara M.. (2009). Direct effects of corticosterone on ATP production by mitochondria from immortalized hypothalamic GT1-7 neurons. J. Steroid Biochem. Mol. Biol. 117, 50–55. 10.1016/j.jsbmb.2009.07.002 19631743

[B16] Guerra G. P. Mello C. F. Bochi G. V. Pazini A. M. Fachinetto R. Dutra R. C.. (2011). Hippocampal PKA/CREB pathway is involved in the improvement of memory induced by spermidine in rats. Neurobiol. Learn. Mem. 96, 324–332. 10.1016/j.nlm.2011.06.007 21708277

[B17] Harrison F. E. Hosseini A. H. McDonald M. P. (2009). Endogenous anxiety and stress responses in water maze and Barnes maze spatial memory tasks. *Behav*. Brain Res. 198, 247–251. 10.1016/j.bbr.2008.10.015 18996418 PMC2663577

[B18] He D. Li T. Yang X. Xu Y. Sun H. (2023). Sudan Black B treatment for reducing autofluorescence in human glioma tissue and improving fluorescent signals of bacterial LPS staining. J. Biophotonics 16:e202200357. 10.1002/jbio.202200357 36633394

[B19] Jekabsons M. B. Nicholls D. G. (2004). In situ respiration and bioenergetic status of mitochondria in primary cerebellar granule neuronal cultures exposed continuously to glutamate^*^. J. Biol. Chem. 279, 32989–33000. 10.1074/jbc.M401540200 15166243

[B20] Johnson B. P. Vitek R. A. Geiger P. G. Huang W. Jarrard D. F. Lang J. M.. (2018). Vital ex vivo tissue labeling and pathology-guided micropunching to characterize cellular heterogeneity in the tissue microenvironment. BioTechniques 64, 13–19. 10.2144/000114626 29384072 PMC5814138

[B21] Kim H. Y. Lee K. Y. Lu Y. Wang J. Cui L. Kim S. J.. (2011). Mitochondrial Ca(2+) uptake is essential for synaptic plasticity in pain. J. Neurosci. 31, 12982–12991. 10.1523/JNEUROSCI.3093-11.2011 21900577 PMC3179262

[B22] Li Z. Okamoto K.-I. Hayashi Y. Sheng M. (2004). The importance of dendritic mitochondria in the morphogenesis and plasticity of spines and synapses. Cell 119, 873–887. 10.1016/j.cell.2004.11.003 15607982

[B23] Medina A. C. Charles J. R. Espinoza-Gonzalez V. Sanchez-Resendis O. Prado-Alcala R. A. Roozendaal B.. (2007). Glucocorticoid administration into the dorsal striatum [corrected] facilitates memory consolidation of inhibitory avoidance training but not of the context or footshock components. *Learn*. Mem. 14, 673–677. 10.1101/lm.654407 17911370

[B24] Miura K. Paul-Gilloteaux P. Tosi S. Colombelli J. (2020). “Workflows and components of bioimage analysis,” in Bioimage Data Analysis Workflows, eds. K. Miura and N. Sladoje (Cham: Springer International Publishing), 1–7. 10.1007/978-3-030-22386-1_1

[B25] Miyoshi E. Wietzikoski E. C. Bortolanza M. Boschen S. L. Canteras N. S. Izquierdo I.. (2012). Both the dorsal hippocampus and the dorsolateral striatum are needed for rat navigation in the Morris water maze. *Behav*. Brain Res. 226, 171–178. 10.1016/j.bbr.2011.09.011 21925543

[B26] Moreira C. G. Barbiero J. K. Ariza D. Dombrowski P. A. Sabioni P. Bortolanza M.. (2012). Behavioral, neurochemical and histological alterations promoted by bilateral intranigral rotenone administration: A new approach for an old neurotoxin. *Neurotox*. Res. 21, 291–301. 10.1007/s12640-011-9278-3 21953489

[B27] Moutsatsou P. Psarra A. M. Tsiapara A. Paraskevakou H. Davaris P. Sekeris C. E. (2001). Localization of the glucocorticoid receptor in rat brain mitochondria. *Arch. Biochem*. Biophys. 386, 69–78. 10.1006/abbi.2000.2162 11361002

[B28] Mu S. OuYang L. Liu B. Zhu Y. Li K. Zhan M.. (2011). Protective effect of melatonin on 3-NP induced striatal interneuron injury in rats. *Neurochem*. Int. 59, 224–234. 10.1016/j.neuint.2011.05.009 21693149

[B29] Mulcahy P. Walsh S. Paucard A. Rea K. Dowd E. (2011). Characterisation of a novel model of Parkinson's disease by intra-striatal infusion of the pesticide rotenone. Neuroscience 181, 234–242. 10.1016/j.neuroscience.2011.01.038 21277943

[B30] National Research Council (2011). Guide for the Care and Use of Laboratory Animals. Washington, DC: National Academy of Sciences.

[B31] NORMA (2001). Oficial Mexicana NOM-62-ZOO-1999. Especificaciones técnicas para la producción, cuidado y uso de los animales de laboratorio. México: SENASICA.

[B32] Paraskevopoulou F. Herman M. A. Rosenmund C. (2019). Glutamatergic innervation onto striatal neurons potentiates GABAergic synaptic output. J. Neurosci. 39, 4448–4460. 10.1523/JNEUROSCI.2630-18.2019 30936241 PMC6554626

[B33] Paxinos G. Watson C. (2007). The Rat Brain in Stereotaxic Coordinates. Burlington, MA: Academic Press.

[B34] Pegueros-Maldonado R. Pech-Pool S. M. Blancas J. J. Prado-Alcalá R. A. Arámburo C. Luna M.. (2024). Inhibition of corticosterone synthesis impairs cued water maze consolidation, but it does not affect the expression of BDNF, CK2 and SGK1 genes in dorsal striatum. Front. Behav. Neurosci. 18:1341883. 10.3389/fnbeh.2024.1341883 38468708 PMC10925660

[B35] Pickrell A. M. Pinto M. Hida A. Moraes C. T. (2011). Striatal dysfunctions associated with mitochondrial DNA damage in dopaminergic neurons in a mouse model of parkinson's disease. J. Neurosci. 31, 17649–17658. 10.1523/JNEUROSCI.4871-11.2011 22131425 PMC3361134

[B36] Quirarte G. L. de la Teja I. S. Casillas M. Serafin N. Prado-Alcala R. A. Roozendaal B. (2009). Corticosterone infused into the dorsal striatum selectively enhances memory consolidation of cued water-maze training. *Learn*. Mem. 16, 586–589. 10.1101/lm.1493609 19794182

[B37] Radad K. Rausch W.-D. Gille G. (2006). Rotenone induces cell death in primary dopaminergic culture by increasing ROS production and inhibiting mitochondrial respiration. *Neurochem*. Int. 49, 379–386. 10.1016/j.neuint.2006.02.003 16580092

[B38] Reul J. M. de Kloet E. R. (1985). Two receptor systems for corticosterone in rat brain: microdistribution and differential occupation. Endocrinology 117, 2505–2511. 10.1210/endo-117-6-2505 2998738

[B39] Rice J. P. Wallace D. G. Hamilton D. A. (2015). Lesions of the hippocampus or dorsolateral striatum disrupt distinct aspects of spatial navigation strategies based on proximal and distal information in a cued variant of the Morris water task. *Behav*. Brain Res. 289, 105–117. 10.1016/j.bbr.2015.04.026 25907746 PMC4441542

[B40] Sandi C. Loscertales M. Guaza C. (1997). Experience-dependent facilitating effect of corticosterone on spatial memory formation in the water maze. Eur. J. Neurosci. 9, 637–642. 10.1111/j.1460-9568.1997.tb01412.x 9153570

[B41] Vázquez-Martínez O. Valente-Godínez H. Quintanar-Stephano A. Gasca-Martínez D. López-Cervantes M. L. Palma-Tirado L.. (2019). Reduced liver lipid peroxidation in subcellular fractions is associated with a hypometabolic state in rats with portacaval anastomosis. Oxid. Med. Cell Longev. 2019:4565238. 10.1155/2019/4565238 30918579 PMC6409024

[B42] Verstreken P. Ly C. V. Venken K. J. T. Koh T.-W. Zhou Y. Bellen H. J. (2005). Synaptic mitochondria are critical for mobilization of reserve pool vesicles at drosophila neuromuscular junctions. Neuron 47, 365–378. 10.1016/j.neuron.2005.06.018 16055061

