# Corrigendum: Differential timing of mitochondrial activation in rat dorsal striatum induced by procedural learning and swimming

**DOI:** 10.3389/fnmol.2025.1586135

**Published:** 2025-03-20

**Authors:** Rogelio Pegueros-Maldonado, Antonio Fuentes-Ibañez, Mónica M. Monroy, Oscar A. Gutiérrez, Norma Serafín, Santiago M. Pech-Pool, Mauricio Díaz-Muñoz, Gina L. Quirarte

**Affiliations:** ^1^Departamento de Neurobiología Conductual y Cognitiva, Instituto de Neurobiología, Universidad Nacional Autónoma de México, Querétaro, Mexico; ^2^Facultad de Química, Universidad Autónoma de Querétaro, Querétaro, Mexico; ^3^Departamento de Neurobiología Celular y Molecular, Instituto de Neurobiología, Universidad Nacional Autónoma de México, Querétaro, Mexico

**Keywords:** cued Morris water maze, consolidation, dorsal striatum, mitochondrial activity, corticosterone, rotenone

In the published article, there was an error in the Funding statement. The funding grant number for Universidad Nacional Autónoma de México was erroneously written as “PAPIIT-UNAM IN209621”, as well as an incorrect name, stated “Consejo Nacional de Ciencia y Tecnología (CONACYT CVU 700765).” The corrected Funding statement appears below.

## Funding

The author(s) declare that financial support was received for the research and/or publication of this article. This work was supported by the Programa de Apoyo a Proyectos de Investigación e Innovación Tecnológica, Dirección General de Asuntos del Personal Académico, Universidad Nacional Autónoma de México (Grants PAPIITUNAM IN209822 and IN207224). RP-M received a scholarship from Consejo Nacional de Humanidades, Ciencias y Tecnologías (CONAHCYT CVU 700765).

The authors apologize for this error and state that this does not change the scientific conclusions of the article in any way. The original article has been updated.

## Publisher's note

All claims expressed in this article are solely those of the authors and do not necessarily represent those of their affiliated organizations, or those of the publisher, the editors and the reviewers. Any product that may be evaluated in this article, or claim that may be made by its manufacturer, is not guaranteed or endorsed by the publisher.

