# Supplementary figures and images for: Differential timing of mitochondrial activation in rat dorsal striatum induced by procedural learning and swimming
Source: Front Mol Neurosci. 2024 Dec 4;17:1495027. doi: 10.3389/fnmol.2024.1495027 (PMC11652596; doi:10.3389/fnmol.2024.1495027)

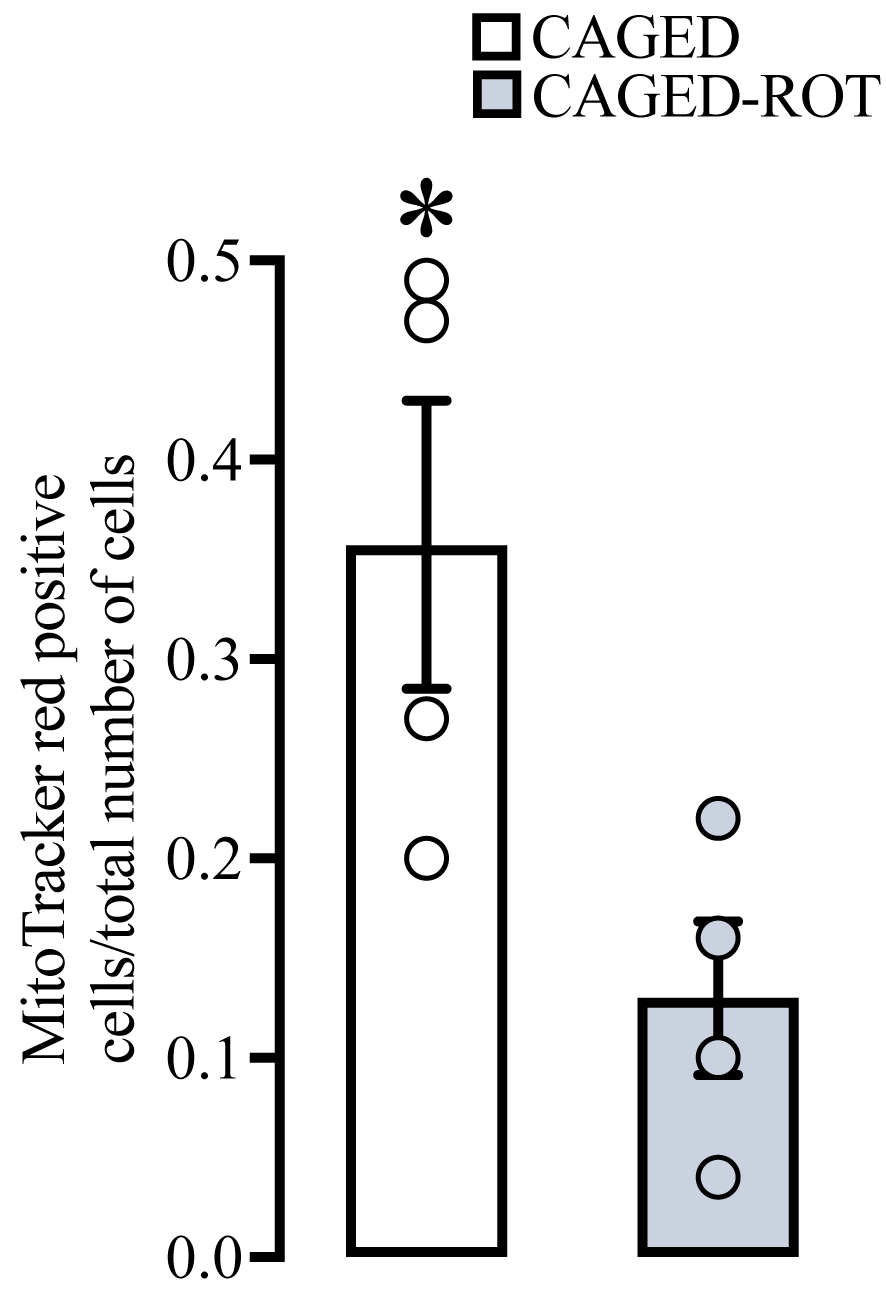

Supplement: Supplementary file 1 [file Image_1.tif]
